# Bioaccessibility and Cellular Uptake of β-Carotene Encapsulated in Model O/W Emulsions: Influence of Initial Droplet Size and Emulsifiers

**DOI:** 10.3390/nano7090282

**Published:** 2017-09-20

**Authors:** Wei Lu, Alan L. Kelly, Song Miao

**Affiliations:** 1 Teagasc Food Research Centre, Moorepark, Fermoy, Cork P61C996, Ireland; wei.lu@teagasc.ie; 2 School of Food and Nutritional Sciences, University College Cork, Cork T12YN60, Ireland; a.kelly@ucc.ie; 3 China-Ireland International Cooperation Center for Food Material Science and Structure Design, Fujian Agriculture and Forestry University, Fuzhou 350002, China

**Keywords:** emulsion, β-carotene, digestion, cellular uptake, bioavailability

## Abstract

The effects of the initial emulsion structure (droplet size and emulsifier) on the properties of β-carotene-loaded emulsions and the bioavailability of β-carotene after passing through simulated gastrointestinal tract (GIT) digestion were investigated. Exposure to GIT significantly changed the droplet size, surface charge and composition of all emulsions, and these changes were dependent on their initial droplet size and the emulsifiers used. Whey protein isolate (WPI)-stabilized emulsion showed the highest β-carotene bioaccessibility, while sodium caseinate (SCN)-stabilized emulsion showed the highest cellular uptake of β-carotene. The bioavailability of emulsion-encapsulated β-carotene based on the results of bioaccessibility and cellular uptake showed the same order with the results of cellular uptake being SCN > TW80 > WPI. An inconsistency between the results of bioaccessibility and bioavailability was observed, indicating that the cellular uptake assay is necessary for a reliable evaluation of the bioavailability of emulsion-encapsulated compounds. The findings in this study contribute to a better understanding of the correlation between emulsion structure and the digestive fate of emulsion-encapsulated nutrients, which make it possible to achieve controlled or potential targeted delivery of nutrients by designing the structure of emulsion-based carriers.

## 1. Introduction

Carotenoids are a class of natural pigments abundant in plants and fruits that can have many health benefits when consumed at proper levels. Previous studies have shown that carotenoids possess strong antioxidant activity and that intake of carotenoid-rich foods was correlated with the reduced risks of several chronic diseases, including cancers, cardiovascular diseases, age-related macular degeneration and cataracts [1,2]. Several potential mechanisms have been proposed to explain these biological activities, e.g., scavenging free radicals and preventing oxidative damage, altering transcription activity or functioning as precursor of vitamin A [3]. β-carotene is a representative member of the carotenoids family and has been widely studied due to its high pro-vitamin A activity. However, extreme water insolubility and instability greatly limit the health benefits of β-carotene. Therefore, the delivery of β-carotene requires an encapsulation and protection mechanism. Emulsions are ideal carriers for lipophilic nutrients, such as β-carotene, due to their ease of operation, maintenance of chemical stability, controlled release and potential for target delivery of encapsulated compounds [4]. 

Since emulsions are widely used as delivery systems for lipophilic nutrients [4,5], an in-depth understanding of the biological fate of emulsion droplets and encapsulated compounds in the digestive tract is necessary for optimizing the delivery efficiency of emulsions. The determination of the changes of droplet properties, e.g., size, surface charge and the subsequent release of encapsulated compounds during digestion, can also contribute to a better understanding of the mechanism of improved bioavailability by emulsion delivery. When being exposed to gastrointestinal tract (GIT) digestion, emulsions can show great changes in their droplet size, surface charge or compositions [6,7], due to the extremely acidic environment in the gastric phase or as a result of enzymatic hydrolysis in the mouth, gastric and intestinal phases; all of these changes can influence the digestion of emulsion droplets and thus the biological fate of nutrients within droplets. 

Many previous studies have investigated the influence of emulsion structure, e.g., droplet size or emulsifiers, on the digestibility of lipid droplets in emulsions [8], the physical and chemical stability of emulsion-encapsulated nutrients [3] and the release of these encapsulated nutrients after passing through simulated GIT digestion [9]. The bioaccessibility of these encapsulated nutrients in emulsions with different initial droplet size [10], emulsifiers [11] and oil compositions [12] was also well evaluated by measuring the content of nutrients in micelle fractions after GIT digestion. However, these studies did not investigate the absorption of these nutrient-loaded micelles by enterocytes, which is important for the evaluation of the bioavailability of encapsulated nutrients. This may also be the main cause of the inconsistency observed between the bioaccessibility and the in vivo bioavailability of emulsion-encapsulated nutrients [13]. In addition, Dairy proteins are widely used as food emulsion stabilizers due to their edibility, health benefits and good amphiphilic properties. Many studies have been done on dairy protein-stabilized emulsions. However, the information on the cellular uptake of encapsulated nutrients in dairy-protein-stabilized emulsions (e.g., whey protein isolate or casein) after passing through GIT was very limited. The comparison between different dairy proteins concerning their influence on the digestion behavior of emulsions containing nutrients in GIT and the subsequent enterocytes cell absorption of released nutrients after GIT still needs further investigation. Furthermore, little is known about the influence of small initial droplet sizes (~1 μm) on the bioaccessibility of encapsulated nutrients. 

Therefore, this study was designed to investigate the bioaccessibility and cellular uptake of an encapsulated lipophilic nutrient (β-carotene) in emulsions with different initial droplet sizes (~1 μm) and emulsifiers (whey protein isolate, sodium caseinate and Tween 80) by the simulated GIT digestion system and the Caco-2 cellular uptake assay. The changes of emulsion properties, such as droplet size and surface charge, during GIT digestion were also tested. 

## 2. Material and Methods 

### 2.1. Materials

β-carotene (BC) (>93%, UV), sodium caseinate (SCN), Tween^®^ 80 (Polysorbate 80, TW80), pepsin (≥250 unit/mg), pancreatin (4× USP), bile salts, Dulbecco’s Modified Eagle’s Medium (DMEM) (containing 4.5 g/L D-glucose), penicillin and streptomycin (100×), fetal bovine serum (FBS), phosphate-buffered solution (PBS) and cell lysis buffer were purchased from Sigma-Aldrich (St. Louis, MO, USA). Sunflower oil was purchased from a local supermarket, and whey protein isolate (WPI) was obtained from Davisco Food International (Le Sueur, MN, USA). All other chemicals and reagents used were of AR-grade and obtained from Sigma-Aldrich. 

### 2.2. Emulsion Preparation 

#### 2.2.1. Preparation of BC-Loaded Emulsions with Different Droplet Sizes

A continuous phase was prepared by dissolving WPI (1.0%, *w*/*w*) in water containing 0.01% (*w*/*w*) sodium azide (anti-bacterial agents). The oil phase was prepared by dissolving BC (0.2%, *w*/*w*) in the sunflower oil (10%, *w*/*w*) at 140 °C for 15 s and then mixed with the continuous phase at a speed of 10,000 rpm for 1 min using an Ultra-Turrax (IKA, Staufen, Germany) followed by further homogenization (APV 1000, SPX Flow Technology, Charlotte, NC, USA) at 20 or 70 MPa. 

#### 2.2.2. Preparation of BC-Loaded Emulsions with Different Emulsifiers

WPI, SCN or TW80 was dispersed (1.0%, *w*/*w*) in water containing 0.01% (*w*/*w*) sodium azide as continuous phases. The subsequent emulsion preparation was performed using the same process mentioned above with high-pressure homogenization at 70 MPa.

#### 2.2.3. Characterization of Droplet Size and Surface Charge

The mean droplet size, and zeta potential of emulsions were determined by dynamic light scattering (DLS) using a laser particle analyser (Nano-ZS, Malvern Instruments, Worcestershire, UK). Emulsions were 1000-fold diluted before testing. 

### 2.3. Rheological Analysis 

Rheological properties of emulsions were determined using an AR 2000 ex rheometer (TA Instruments, Crawley, UK)). A concentric cylinder geometry (stator inner radius = 15 mm, rotor outer radius = 14 mm, gap = 5920 μm) were selected. A viscosity test was performed over a shear rate range of 0–200 s^−1^ at 25 °C.

### 2.4. Creaming Stability

The creaming stability of different emulsions was evaluated using a Lumisizer (LUM GmbH, Berlin, Germany) as described previously [14]. In this study, emulsions were centrifuged at 2300× *g* at 25 °C with a scanning rate of once every 10 s for 1200 s. 

### 2.5. In Vitro Simulated GIT Digestion

An in vitro simulated GIT digestion method employed in a previous study [7] was used to digest emulsions. The digesta after each phase (mouth, gastric and intestinal phase) were sampled for the determination of droplet size and zeta potential. The simulated saliva fluid (SSF), gastric fluid (SGF) and intestinal fluid (SIF) were prepared as described previously [7].

Mouth phase: Emulsions were mixed with SSF (1:1, *v*/*v*), and the pH was adjusted to 6.8 and incubated at 37 °C for 10 min with continuous agitation at 100 rpm.

Gastric phase: The digesta from the mouth phase were mixed with the SGF (1:1, *v*/*v*), and the pH of the mixture was adjusted to 2.5. The mixture was then incubated at 37 °C for 2 h with continuous agitation at 100 rpm. The enzyme activity of pepsin in the final mixture was 2000 U/mL.

Small intestinal phase: The digesta sample from the gastric phase was mixed with the SIF (1:1, *v*/*v*). The pH of the mixture was adjusted to 7.0, and it was incubated at 37 °C for 2 h with continuous agitation at 100 rpm. The enzyme activity of pancreatin (based on trypsin) in the final mixture was 100 U/mL.

### 2.6. In Vitro Bioaccessibility of BC

The bioaccessibility of BC after the intestinal phase was evaluated as described previously with minor modification. An aliquot of raw digesta from the intestinal phase was centrifuged at 2700× *g* for 40 min at 4 °C, and the supernatant was collected and considered as the micelle fraction, in which the bioactive compound is solubilized. Aliquots of 2 mL of the raw digesta or the micelle fraction were extracted twice with ethanol/*n*-hexane. The top layer containing the solubilized BC was collected and analysed by RP-HPLC as described below.

The bioaccessibility of encapsulated BC was calculated using the following equation:

(1) $$\mathrm{Bioaccessibility}\;(\%)\;=\;\frac{C_{\mathrm{micelle}}}{C_{\mathrm{initial}}}\;\times 100\%$$

where *C*_micelle_ and *C*_initial_ are the concentration of BC in the micelle fraction after intestinal phase digestion and initial emulsion before GIT digestion, respectively. 

### 2.7. Cellular Uptake by Caco-2 Cells

Caco-2 cells were seeded in a 6-well plate at a density of 3.5 × 10^5^ cells well^−1^, and cellular uptake experiments were performed 5–7 days after seeding. Micelle fractions of different BC-loaded emulsions after the intestinal phase were 20-fold diluted with complete medium. One millilitre of diluted samples was added to each well in a 6-well plate, which was then incubated at 37 °C and 5% CO_2_ for 4 h. Before collection, cells were washed three times with PBS buffer solution. Then, cells were collected, lysed, extracted and analysed for BC content by RP-HPLC. 

### 2.8. Extraction of BC 

BC was extracted from the micelle fraction or raw digesta emulsion systems with ethanol/*n*-hexane (1:2, *v*/*v*) two times. The hexane layers were combined and dried under a stream of nitrogen gas and dissolved in 0.6 mL ethanol for HPLC analysis.

### 2.9. HPLC Analysis of BC

The concentration of BC was determined using an Agilent 1200 series system with a DAD UV-Vis detector (Agilent, Santa Clara, CA, USA); the column was reversed phase C_18_ (4.6 × 250 mm, 5 μm, 300 Å, Phenomenex); the operation temperature was 30 °C; elution was performed with 90% ethanol and 10% acetonitrile from 0 to 15 min; the flow rate was 1 mL/min; the detection wavelength was 450 nm; the injection volume was 20 μL. The peak area of BC on HPLC showed a good linear correlation with the BC concentration in the range of 0.05~5 μg/mL (data not shown). 

### 2.10. Statistical Analysis 

All experiments were repeated at least three times. One-way analysis of variance (ANOVA) was employed to compare means of data. A *t*-test was used to determine the differences between means, and significant differences were determined at the 0.05 level (*p* < 0.05). 

## 3. Results and Discussion 

### 3.1. Characterization of Emulsions

Emulsions showed a reduced droplet size with increasing homogenization pressure (HP) used during their preparation (Table 1), which was as observed in many previous studies [15], and no significant difference in droplet size of emulsions stabilized by whey protein isolate (WPI), sodium caseinate (SCN) and Tween^®^ 80 (TW80), processed at similar homogenization pressures, was observed. Droplets of WPI- and SCN-stabilized emulsions were negatively charged, which is mainly attributed to the protein molecules being negatively charged at pH (7.0), which is higher than their isoelectric point (pH 4.0–5.0). WPI-stabilized emulsions with different droplet sizes (produced at different homogenization pressures) showed similar surface charges. Droplets of TW-stabilized emulsion were also negatively charged, but showed a much lower zeta potential (−25 mV) than that of the emulsions stabilized with proteins (around −53 mV). 

All emulsions showed very low viscosity. The SCN emulsion showed the highest viscosity, followed by WPI- and TW80-stabilized emulsions, respectively (Table 1). WPI emulsions with large or small droplets did not significantly differ in viscosity. The viscosity of emulsions can be influenced by the proportion of the oil phase and emulsifiers [16,17] and increases with increasing oil content, owing to the increased interfacial tension with water [18]. 

**Figure 1:**
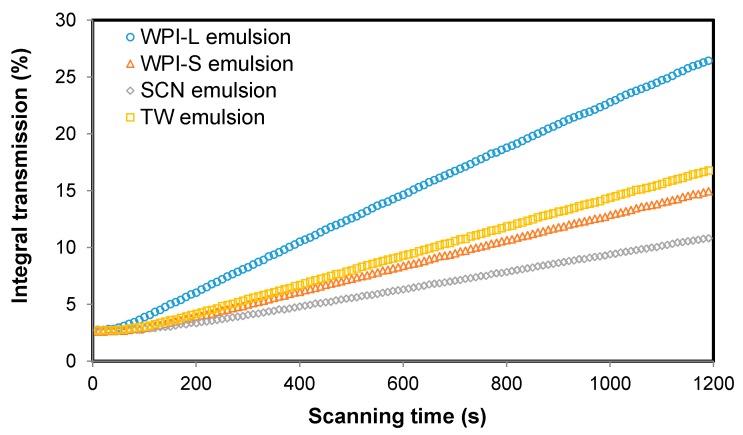
Integral light transmission of different emulsions. WPI-L and WPI-S indicate emulsions stabilized by whey protein isolate with large and small droplet sizes, respectively. SCN and TW emulsions indicate emulsions stabilized with sodium caseinate and Tween^®^ 80, respectively.

The SCN-stabilized emulsion showed the best creaming stability (*p* < 0.01), followed by WPI- and TW-stabilized emulsion, respectively (Figure 1). The WPI-stabilized emulsion with a small droplet size showed better creaming resistance than that with a large droplet size (*p* < 0.01). These results suggested that the creaming stability of emulsions is dependent on their initial droplet size and interfacial composition. 

**Figure 2:**
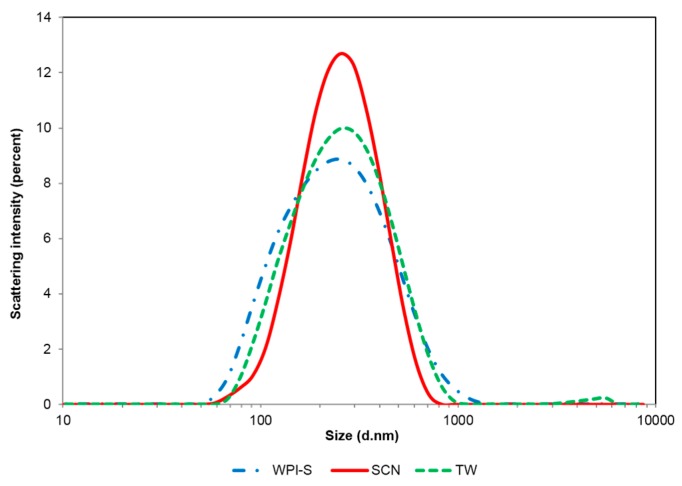
Size distribution of emulsions with different emulsifiers. WPI-S indicates whey protein isolate-stabilized emulsion with small droplet size; SCN indicates sodium caseinate-stabilized emulsion; TW indicates Tween^®^ 80-stabilized emulsion.

According to Stokes’ law, creaming velocity (*V*) is related to the radius of the particle (*R*), the viscosity (*μ*) and density of the particle ($\rho_{p}$) and the continuous phase ($\rho_{f}$). Emulsions with smaller droplet sizes, higher viscosity or higher particle density are thus expected to show better creaming stability. In this study, the SCN-stabilized emulsion showed higher viscosity and a narrower size distribution (Figure 2), as well as a lower PdI (Table 1) than WPI and TW emulsions, which may explain why the former emulsion showed the best creaming stability.

### 3.2. Characterization of Emulsions after Being Exposed to GIT Digestion 

Exposure to GIT digestion can result in great changes in the properties of emulsions, e.g., droplet size and surface charge, which accordingly will influence the digestion and absorption of nutrients incorporated into emulsions. Thus, the droplet size and surface charge of BC-loaded emulsions after being exposed to GIT were investigated. 

All emulsions showed only a slight increase in droplet size after exposure to simulated mouth digestion (Table 2). This is mainly attributed to the absence of mucin from the SSF used in this study because mucin is the main cause of the increase in droplet size during mouth-phase digestion [10].

**Figure 3:**
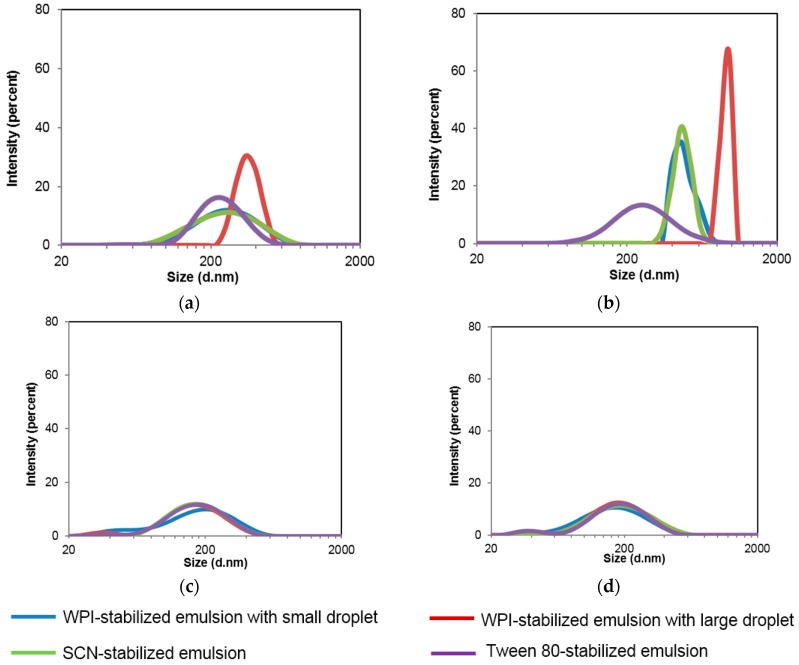
Size distribution of emulsions after passing through simulated GIT digestion. (**a**) Mouth phase; (**b**) Gastric phase; (**c**) Intestinal phase; (**d**) Micelle fractions. WPI-S and WPI-L indicate whey protein isolate-stabilized emulsions with small and large droplet sizes, respectively; SCN indicates sodium caseinate-stabilized emulsion; TW indicates Tween^®^ 80-stabilized emulsion.

After the gastric phase, a dramatic increase in average droplet size (Table 2) and size distribution (Figure 3b) of all emulsions was observed, except for the TW80-stabilized emulsion. The WPI-stabilized emulsion showed a larger average droplet size (774 nm) at this point than that of the SCN-stabilized emulsion (747 nm). The WPI-stabilized emulsion with large initial droplets showed a larger droplet size (1256 nm) than that with the small initial droplets (774 nm). The results suggested that the initial emulsion structure, e.g., emulsifiers and droplet sizes, can greatly influence the properties after being exposed to simulated gastric digestion. The dramatic increase in droplet size during this process is potentially attributed to several factors, including the low pH, incubation at 37 °C, ionic strength and the hydrolysis of interfacial proteins by pepsin. However, incubation at 37 °C for 2 h did not increase the droplet size of WPI- and SCN-stabilized emulsions (data not shown), and the previous study also confirmed that dairy protein-stabilized emulsions were stable at pH < 4.0 [19]. Mao et al. [20] found that WPI-stabilized multilayer emulsion droplets aggregated significantly in a NaCI solution of strength ≥150 mM because the relatively high ion strength can potentially reduce the electrostatic repulsion between droplets [21] and lead to their aggregation. Furthermore, pepsin in SGF can hydrolyse WPI and SCN at the oil-water interface and result in partially break-down of the interfacial layer structure and, thus, the aggregation of oil droplets. These findings suggest that the increased droplet size of emulsions during the gastric phase digestion may be mainly induced by the ionic strength (177 mM) in SGF and the hydrolysis of proteins at the interface by pepsin. 

Compared with the gastric phase, the droplet size of all emulsions dramatically decreased after the intestinal phase (Table 2). The WPI-stabilized emulsion showed the smallest average droplet size of 148 nm after intestinal phase digestion, followed by TW- (157 nm) and SCN-stabilized (166 nm) emulsions, respectively. No significant difference between the WPI-stabilized emulsion with small and large initial droplet sizes was observed. The decrease in droplet size was mainly attributed to the rapid break-down of droplets due to the hydrolysis of proteins (WPI and SCN) and Tween 80 by trypsin and lipase, respectively, and the subsequent formation of small micelles stabilized by bile salts (Figure 3c,d). 

All emulsions were negatively charged after mouth phase digestion, which is mainly attributed to the protein emulsifiers (WPI and SCN) being negatively charged at pH 6.8, which is above their isoelectric point (pI). The SCN-stabilized emulsion had the highest surface charge, of −55.1 mV, followed by the WPI- and TW-stabilized emulsions, at −51.7 mV −24.3 mV, respectively. No significant difference in surface charge between WPI-stabilized emulsions with small (−51.7 mV) and large (−53.3 mV) droplet sizes was observed (Table 2). 

WPI- and SCN-stabilized emulsions were positively charged after the gastric phase (Table 2), as expected because pH 2.5 is below their pI. The WPI-stabilized emulsion had a higher surface charge than the SCN emulsion (9.0 mV) after the gastric phase, and the WPI-stabilized emulsion with small initial droplets showed a higher surface charge (17.6 mV) than the emulsion with a large initial droplet size (11.1 mV). The TW-stabilized emulsion was almost neutrally charged after the gastric phase. 

After the intestinal phase, all emulsions were negatively charged, and there was no significant difference in charge between different emulsions. This is mainly attributed to the enzymatic hydrolysis of proteins (WPI and SCN) and TW at the droplet surface by trypsin and lipase and the subsequent absorption of other anionic molecules, e.g., bile salts, to the droplet/micelle surface, resulting in a uniformly negatively-charged surface [10]. 

### 3.3. In Vitro Bioaccessibility of BC 

Effects of droplet size and the selection of emulsifiers on the in vitro bioaccessibility of emulsion-encapsulated BC were investigated, as emulsion structure and interfacial composition can significantly influence the bioaccessibility of nutrients incorporated into emulsions [10,12].

**Figure 4:**
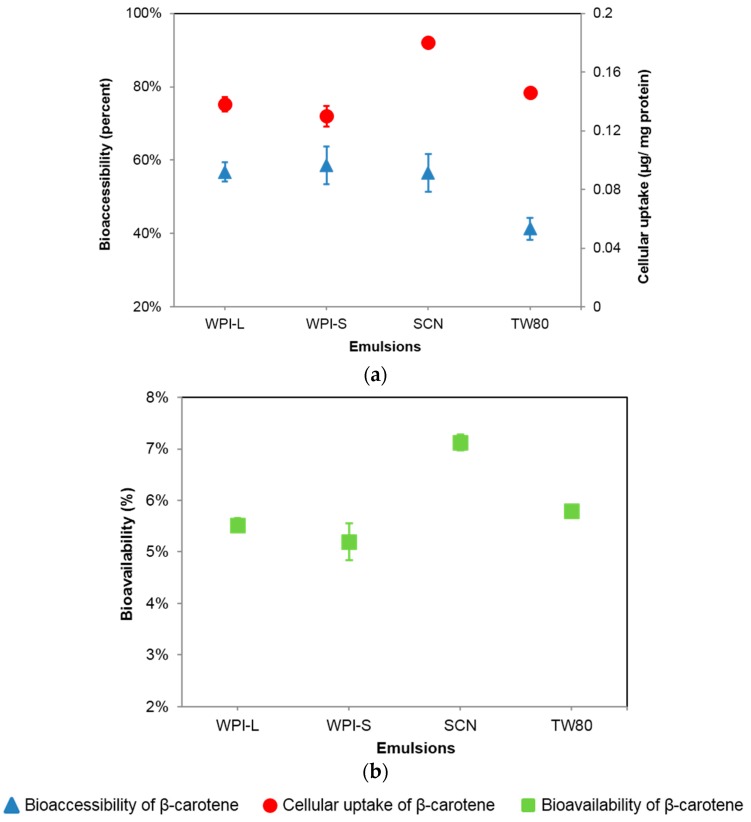
(**a**) Bioaccessibility and cellular uptake of encapsulated β-carotene; (**b**) Bioavailability of encapsulated β-carotene based on the results of bioaccessibility and cellular uptake. WPI-S and WPI-L indicate whey protein isolate-stabilized emulsions with small and large droplet sizes, respectively; SCN indicates sodium caseinate-stabilized emulsion; TW indicates Tween^®^ 80-stabilized emulsion.

The WPI-stabilized emulsion (WPI-S) showed the highest (*p* < 0.05) bioaccessibility of 58.5%, followed by the SCN- and TW-stabilized emulsions of 56.5% and of 41.3%, respectively (Figure 4a). No significant difference between WPI-stabilized emulsions with small (WPI-S) and large initial droplet sizes (WPI-L) was observed. This may be mainly attributed to the initial droplet size in this study (*d* < 0.4 μm) being not as large as in previous studies [10,22], in which significant differences in the bioaccessibility of emulsion-encapsulated nutrients in large and small droplets were observed. However, when the initial droplet size was below 1 μm, this difference becomes less significant. 

Generally, the bioaccessibility of emulsion-encapsulated nutrients is closely related to the structure of the emulsion, including initial droplet size, emulsifiers or oil phase compositions and proportions. TW can be hydrolysed by lipase [23] in intestinal phase digestion and can act as a competitive substrate with lipid inside the oil droplets, which accordingly may decrease the hydrolysis rate of oil and thus potentially decrease the release of encapsulated BC. This may explain why the TW-stabilized emulsion showed a lower bioaccessibility than those stabilized with WPI and SCN.

### 3.4. Cellular Uptake of BC

In order to further evaluate the bioavailability of emulsion-encapsulated BC, to understand why there is an inconsistency between the results of bioaccessibility by measuring the content of nutrients in micelle fractions after GIT and in vivo bioavailability, a Caco-2 cell culture assay was employed to investigate the cellular uptake of BC after GIT. 

The SCN-stabilized emulsion showed a significantly higher (*p* < 0.05) cellular uptake of BC (0.180 μg/mg protein) than TW80- (0.146 μg/mg protein) and WPI-stabilized (0.130 μg/mg protein) emulsions (WPI-S) (Figure 4a), which is obviously different with the results of bioaccessibility described above. This may explain why an inconsistency between the results of in vitro bioaccessibility and in vivo bioavailability was observed. Generally, increased cellular uptakes of nanoparticles are mainly attributed to their reduced particle size and different surface structures. However, the micelle fraction of SCN-stabilized emulsion showed even a larger average droplet size than that of WPI- and TW-stabilized emulsions and the surface charge of all of the micelles was not significantly different (*p* > 0.1) (Table 3), indicating that increased cellular uptake of encapsulated BC in the SCN-stabilized emulsion could not be attributed to the droplet size and surface charge. 

As is known, casein shows better surface activity than whey proteins (α-lactalbumin and β-lactoglobulin) [24], which is mainly attributed to their different amino acid sequences. After hydrolysis by pepsin and trypsin, SCN may produce more peptides that have amphiphilic structures than WPI, and these peptides can bind to the surface of newly-formed BC-loaded micelles, facilitating the interaction of micelles with Caco-2 cells and, thus, increasing the cellular uptake of BC. This may explain why SCN-stabilized emulsion showed a higher cellular uptake of BC than WPI-stabilized emulsion in this study.

No significant difference between WPI- and TW-stabilized emulsions was observed. Although WPI-stabilized emulsions with different initial droplet sizes showed significantly different micelle sizes after intestinal phase digestion, also no significant difference in cellular uptake of BC was observed between them. 

Based on the results of in vitro bioaccessibility and cellular uptake, the bioavailability of BC in this study can be calculated according to the following equation [25]:

Bioavailability (%) = *F_C_* × *F_B_* × *F_A_*× *F_M_* where *F_C_* is the fraction of BC before passing through GIT; *F_B_* is the bioaccessibility, which is the fraction of BC in micelles after intestinal phase digestion in this study; *F_A_* is the absorption, which is the cellular uptake fraction of BC in this study; *F_M_* is the metabolism, which is the fraction of BC in a bioactive form after the metabolism within GIT, epithelium cells, blood circulation system or liver. This study did not refer to the test of metabolism within blood circulation and liver. Hence, *F_M_* was not used in the calculation of the bioavailability. 

As is shown in Figure 4b, the results of the bioavailability of different emulsions showed the same variation tendency with the results of cellular uptake (Figure 4a). No significant difference in bioavailability of BC between WPI-stabilized emulsions with large and small droplet sizes was observed. SCN-stabilized emulsion showed the highest of 7.2%, followed by TW80- and WPI-stabilized emulsions of 5.8% and 5.2%, respectively, which showed the same order as the results of cellular uptake of BC (Figure 4b), indicating that increased bioavailability of these emulsion-encapsulated nutrients may be mainly attributed to their increased cellular uptake of nutrient-loaded micelles after passing through GIT. The cellular uptake assay is accordingly considered as a necessary assay for a better evaluation of the in vitro bioavailability of encapsulated nutrients. 

## 4. Conclusions

The choice of emulsifier, between whey protein isolate (WPI), sodium caseinate (SCN) and Tween 80 (TW), significantly influenced the creaming stability, surface charge and viscosity of β-carotene-loaded emulsions. The SCN-stabilized emulsion showed the highest creaming stability and viscosity in all emulsions. Passing emulsions through simulated GIT led to great changes in their droplet size, surface charge and compositions, and these changes were dependent on initial droplet sizes and interfacial compositions. However, in vitro bioaccessibility and cellular uptake of encapsulated β-carotene after GIT were mainly dependent on the interfacial compositions (emulsifiers). The SCN-stabilized emulsion showed the highest cellular uptake of β-carotene, followed by TW80- and WPI-stabilized emulsions, respectively, which showed the same order as the results of the bioavailability of β-carotene, potentially indicating that the increased bioavailability of emulsion-encapsulated β-carotene is mainly attributed to their increased cellular uptake. In addition, an inconsistency between the results of the in vitro bioaccessibility and bioavailability of β-carotene was observed, which may be the main cause of the reported inconsistency between the results of the in vitro bioaccessibility and in vivo bioavailability of emulsion-encapsulated nutrients, suggesting that the cellular uptake assay is necessary for a reliable evaluation of the in vitro bioavailability and may be useful for predicting the in vivo bioavailability of emulsion-encapsulated compounds. 

## Figures and Tables

**Table 1:** Droplet size, zeta potential, polydispersity index (PdI), viscosity and creaming index of emulsions.

Emulsions	Size (d nm)	Zeta Potential (mV)	Polydispersity Index (PdI)	Viscosity (mPa·s)	Creaming Index
WPI-L	472 ± 20 ^a^	−53.2 ± 1.7 ^a^	0.24 ± 0.07 ^a^	1.78 ± 0.02 ^b^	0.327 ± 0.007 ^a^
WPI-S	205 ± 4 ^b^	−52.7 ± 0.6 ^a^	0.24 ± 0.03 ^a^	1.76 ± 0.02 ^b^	0.169 ± 0.003 ^c^
SCN	223 ± 12 ^b^	−52.1 ± 0.7 ^a^	0.18 ± 0.02 ^b^	1.94 ± 0.02 ^a^	0.111 ± 0.002 ^d^
TW	227 ± 12 ^b^	−25.1 ± 0.5 ^b^	0.22 ± 0.01 ^a^	1.72 ± 0.02 ^b^	0.193 ± 0.005 ^b^

WPI-L and WPI-S indicate emulsions stabilized by whey protein isolate with large and small initial droplet sizes; SCN and TW indicate emulsions stabilized by sodium caseinate and Tween^®^ 80. Different superscript letters indicate significant differences between values in a column (*p* < 0.05).

**Table 2:** Particle size and surface charge of emulsions after being exposed to simulated GIT digestion.

Emulsion	Droplet Size (d nm)			Zeta Potential (mV)			Polydispersity Index (PdI)		
	Mouse Phase	Gastric Phase	Intestinal Phase	Mouse Phase	Gastric Phase	Intestinal Phase	Mouse Phase	Gastric Phase	Intestinal Phase
WPI-S	224 ± 11 ^b^	774 ± 16 ^b^	148 ± 12 ^a^	−51.7 ± 0.6 ^a^	17.6 ± 0.9 ^a^	−64.3 ± 7.0 ^a^	0.20 ± 0.02 ^b^	0.71 ± 0.03 ^b^	0.38 ± 0.01 ^a^
WPI-L	471 ± 11 ^a^	1256 ± 242 ^a^	153 ± 9 ^a^	−53.3 ± 1.6 ^a^	11.1 ± 0.5 ^b^	64.0 ± 0.4 ^a^	0.31 ± 0.09 ^a^	1.0 ± 0.00 ^a^	0.32 ± 0.04 ^a^
SCN	224 ± 13 ^b^	747 ± 20 ^b^	166 ± 8 ^a^	−55.1 ± 0.4 ^a^	9.0 ± 0.5 ^b^	−60.5 ± 3.3 ^a^	0.19 ± 0.00 ^b^	0.70 ± 0.07 ^b^	0.23 ± 0.00^c^
TW80	229 ± 6 ^b^	233 ± 8^c^	157 ± 9 ^a^	−14.3 ± 0.7 ^b^	0.51 ± 0.0^c^	−62.1 ± 1.0 ^a^	0.16 ± 0.04 ^b^	0.19 ± 0.01^c^	0.29 ± 0.04 ^b^

WPI-L and WPI-S indicate emulsions stabilized by whey protein isolate with large and small droplets, respectively; SCN and TW indicate emulsions stabilized with sodium caseinate and Tween^®^ 80, respectively. Different superscript letters indicate significant differences between values in a column (*p* < 0.05).

**Table 3:** Particle size and zeta potential (ZP) of micelle fractions from different emulsions and the in vitro bioavailability and cellular uptake of encapsulated β-carotene after passing through GIT (mean ± STD, *n* = 2).

Micelles	Size (d nm)	ZP (mV)
WPI-L	158 ± 3 ^a^	−65.0 ± 0.5 ^a^
WPI-S	142 ± 6 ^b^	−64.2 ± 0.7 ^a^
SCN	160 ± 10 ^a^	−61.1 ± 3.3 ^a^
TW	156 ± 7 ^a^	−63.0 ± 1.0 ^a^

WPI-L and WPI-S indicate micelles from emulsions stabilized by whey protein isolate with large and small droplet sizes after GIT, respectively; SCN and TW indicate micelles from sodium caseinate- and Tween^®^ 80-stabilized emulsions after GIT, respectively. Different superscript letters indicate significant difference between values in a column (*p* < 0.05).
